# 

**Published:** 1831-02

**Authors:** 


### MONTHLY LIST OF MEDICAL BOOKS.

[*Medical Works cannot be entered on this List unless a copy be sent for the purpose, the titles of Books having frequently been sent to us as published, which have not been published for weeks, or even months, after.*]

Monthly Gazette of Practical Medicine. January 1831.

A Manual of Surgery, founded upon the Principles and Practice lately taught by Sir A. Cooper, Bart. F.R.S. &c. and Joseph H. Green, Esq. F.R.S. &c. Third Edition, much enlarged, containing many additional Notes from the Writings of other distinguished Surgeons. Edited by Thomas Castle, F.L.S. of Queen's College, Oxford, &c.—12mo. pp. 515. London: Cox, 1831.

This is precisely the kind of work which the student should peruse before he enters upon more elaborate systems of surgery. The leading principles of the science are described in a very clear manner. Mr. Castle's object is to assist beginners; but, in justice to him, we must observe, that his Manual may be studied with great advantage by practitioners as well as pupils. It is one of the most useful elementary surgical works hitherto published. Some opinions have attracted our notice, which we think require further explanation or modification. In describing the treatment to be observed *after* the operation of strangulated hernia, it is said, "The horizontal posture must be strictly ordered; and, in five or six hours after the operation, give a little sulphate of magnesia, or castor oil: *the more motions your patient has, the better.* You must not imagine, because the patient has two or three motions in the twenty-four hours after the operation, that he will do well; for this is generally the case: *you must, therefore, keep up a free dis-*

*charge from the bowels,* or the next day you will find them bound, the abdomen tense, and tender to the touch, and vomiting will come on. He is at this time in the greatest danger: you must bleed largely, and purge him freely by medicines and injections." We strongly doubt the propriety of such very *free* purging, in *all* cases, after the operation for strangulated hernia. In some instances, we think the practice must be positively dangerous, in many unnecessary. Upon the subject of piles, we observe a practical direction, with which we cannot agree. The ligature is, we believe, properly preferred to excision: but the mode in which the ligature is recommended to be applied strikes us as objectionable. "The application of a ligature is exceedingly painful, if it be drawn tightly: *it should only be applied so as to interrupt the circulation, and destroy the life of the part, without exciting much pain.*" The danger to be feared from the use of the ligature in cases of piles, is great constitutional disturbance, or even tetanus. But our own experience tells us, that the risk is diminished by drawing the ligature as tight as possible, so as entirely to cut off the circulation, and ensure the rapid destruction of the part. We admit, that the pain produced by this mode of operating is considerable, but it is of short continuance, and is generally to be subdued by the free exhibition of opiates. If the ligature be less firmly applied, the operation will probably fail altogether in destroying the part; and the constitutional irritation which arises will be nearly as severe, and much more lasting, than if the ligature is drawn very tight at the time of the operation.

No. I. (to be continued monthly,) of Medical Zoology and Mineralogy; or, Illustrations and Descriptions of the Animals and Minerals employed in Medicine, and of the Preparations derived from them: comprising their generic and specific Characters; English, Provincial, and Foreign Appellations; a copious List of Synonymes; Natural History; Physical, Chemical, and Medical Properties, and Uses: including also a Popular and Scientific Account of Animal, Mineral, Atmospheric, and Gaseous Poisons; with Figures coloured from Nature. Intended to serve as a Continuation or Supplement to the Author's and other Works, on "Medical Botany," and Materia Medica. By John Stephenson, m.d. f.l.s.—London: Wilson.

As far as we can judge from the first number, this work will contain much necessary instruction for medical students.

An Essay on the Powerful Influence of the Spinal Nerves over the Sexual Organs, and through them upon the general State of the Body. Being an Appendix to the First Number of the Monthly Gazette of Practical Medicine. By Edward Harrison, m.d. Ed. f.r.a.s.—8vo. pp. 23. London: Simpkin and Marshall, 1831.

The Medical Annual for 1831; containing a Popular Account of all the new Discoveries in Medicines, and of Domestic Articles of real Utility: forming a complete Modern Dispensatory; with a Selection of Prescriptions of established Efficacy; a Catalogue of Diseases, with the Modes of Treatment (Medical and Dietetic,) which ample Experience has proved to be the most successful; a List of Drugs, with their Doses to Children and Adults; numerous Mechanical Auxiliaries to Medicine, &c. By R. Reece, m.d. &c.—Pp. 124. London: Simpkin and Co. 1831.

Parts V. and VI. Illustrations of Mr. S. Cooper's Surgical Dictionary. Monthly.—Longman, 1831. Containing, in each Part, four Lithographic Plates, with Letter-press Descriptions, and References to the Text.

We would recommend surgical students to purchase these illustrations: by their assistance, the surgical pathology of diseases wiil be more clearly comprehended, and more firmly impressed upon the memory. If the descriptive text were opposite each plate, it would be more convenient for reference.

The Companion to Post-mortem Examinations. Illustrated by six Plates. —Rose, 1831.

We cannot conscientiously promote the introduction of this "Companion" to our friends: it is a trifling and useless work.

### NOTICES.

*Communications have been received from* Dr. Ashburner, Dr. A. T. Thomson, Dr. Barry, &c.

